# Factors affecting academic performance of college students in China during COVID-19 pandemic: a cross-sectional analysis

**DOI:** 10.3389/fpsyg.2023.1268480

**Published:** 2023-11-03

**Authors:** Changle Li, Lili Kang, Toni P. Miles, M. Mahmud Khan

**Affiliations:** ^1^School of Health Management, Fujian Medical University, Fuzhou, China; ^2^College of Public Health, University of Georgia, Athens, GA, United States; ^3^Rosalynn Carter Institute for Caregivers, Atlanta, GA, United States

**Keywords:** health status, Internet use, study time, academic performance, COVID-19 pandemic

## Abstract

**Introduction:**

Understanding the factors that affected academic performance of students during the COVID-19 pandemic will help design effective interventions for improving students’ academic performance during emergency situations as well as during regular academic environment. This cross-sectional study aimed to identify the factors that explain academic performance of students in China during the pandemic.

**Methods:**

Data on college students from the 2020 China Family Panel Studies were used, and the final sample consisted of 728 students. Ordered probit regression models were estimated to explain students’ relative performance in the semester when the in-person classes were suspended by using various student and household-related variables and characteristics. To compute missing values in selected variables, a multiple imputation technique was applied.

**Results:**

The odds of poor academic performance declined with higher Internet use for academic purposes, but Internet use for entertainment increased the probability of being in the poor academic performance. College students who spent more time studying on college work were less likely to have poor academic performance.

**Discussion:**

This study identified the factors (Internet use and study time) associated with academic performance among Chinese college students during the COVID-19 pandemic. These results can be used to design policies to improve educational outcomes and to address educational inequalities.

## 1. Introduction

The World Health Organization (WHO) declared the Coronavirus Disease 2019 (COVID-19) outbreak as a global pandemic on 11 March 2020 (World Health Organization, 2020). Although health was the rationale for this declaration, the pandemic also shut down traditional, face-to-face approaches to educational activities (Adedoyin and Soykan, 2020). To protect the health of its citizens, the Chinese government launched an emergency policy for educational institutions entitled ‘Suspend Classes but Learning Continues’. This policy required both educators and learners to utilize online teaching and learning strategies while all schools were declared closed (Zhang et al., 2020; Gu and Li, 2022). As a result, all colleges and universities delayed the start date of the spring semester and switched to online teaching and learning (Gu and Li, 2022). Fortunately, with the continued expansion and development of Internet technology and the popularity of mobile devices in China, most students should have access to digital learning technology. However, switching under these circumstances has been framed as emergency remote teaching and it comes with benefits and challenges (Hodges et al., 2020).

In China, face-to-face teaching is the preferred and widely used approach. The COVID-19 pandemic has forced college students to adapt to online learning for a broader array of academic subjects. The COVID-19 pandemic increased anxiousness about the uncertainty associated with the disease. Social distancing and lockdowns led to fewer learning opportunities through social interactions like study groups (Chu and Li, 2022). The technological aspects of distance teaching and learning were not the only barriers in the participation of students in academic activities. Sudden outbreaks make college students more prone to change in health-related behaviors (Gonzalez-Ramirez et al., 2021; Tran et al., 2021; Gadi et al., 2022), physical health condition (Gestsdottir et al., 2021; Gewalt et al., 2022), and psychological stress (Azmi et al., 2022; Rutkowska et al., 2022). The impact of these adverse health situations affected the academic performance of students (Ding et al., 2009; Matingwina, 2018). However, to date, few studies have examined the relationship between health status and academic performance among college students during the COVID-19 pandemic.

By necessity the pandemic lockdowns, increased time spent using the Internet. Usage increased among college students. For students, using the Internet was especially important for online learning as well as for entertainment and social contacts (Fernandes et al., 2021; Burkauskas et al., 2022). However, large amount of time spent on the Internet can be problematic for some individuals as they tend to spend many hours on activities not directly related to learning (Jahan et al., 2021). One study reported a negative and statistically significant association between hours spent online and academic performance of college students (Englander et al., 2010). Most of the previous studies examined the relationship between internet addiction and academic performance among college students. The findings are mixed on how internet addiction affects academic performance (Akhter, 2013; Usman et al., 2014). Few studies employed internet use patterns (for academic and entertainment purposes) in empirical analysis but this study was able to consider this aspect explicitly.

The shutdown of various activities during the COVID period implies that time allocation pattern of individuals had to adjust. With the lowering of time spent in traveling and attending classes, available time must be redistributed. A survey found that 55% of students reported spending less time on learning activities “on their own” during the COVID-19 pandemic, and 18% of students reported spending more time (Gao et al., 2021). Spending more time on learning activities is effective if it capitalizes on use of the time learning takes place during the time, should improve academic performance (Plant et al., 2005), but no conclusive evidence was found between study time and academic performance during the COVID-19 period (Nonis and Hudson, 2010). One study reported a relatively stable ‘inverted U’ relation between study time and academic performance, implying that exceeding optimal times would hinder academic performance (Yang and Zhao, 2021). The findings are mixed on how study time affects academic performance. Teaching and learning evaluations could benefit students with strategies to manage time.

In summary, physical, psychological, and pedagogical components are essential for ‘effective learning’ (Closs et al., 2022). ‘Effective learning’ in an online format is not simply spending time studying online materials and listening to lectures. Online learning requires greater self-direction and self-regulation to achieve academic goals, especially during a period when most activities are under lockdown (Goulão and Menedez, 2015). Understanding the factors that affected academic performance during the COVID-19 pandemic will help design effective interventions for improving students’ academic performance in an online setting and during emergency situations. The purpose of this paper is to identify the factors that explain academic performance of college students in China during the pandemic.

This study is an empirical analysis using nationally representative survey data. The specific research questions of the study are:

*Q1*: How did health status affect academic performance of college students in China during the COVID-19 pandemic?

*Q2*: How did internet use affect academic performance of college students in China during the COVID-19 pandemic?

*Q3*: How did study time affect academic performance of college students in China during the COVID-19 pandemic?

## 2. Methods

### 2.1. Conceptual model

**Figure 1:**
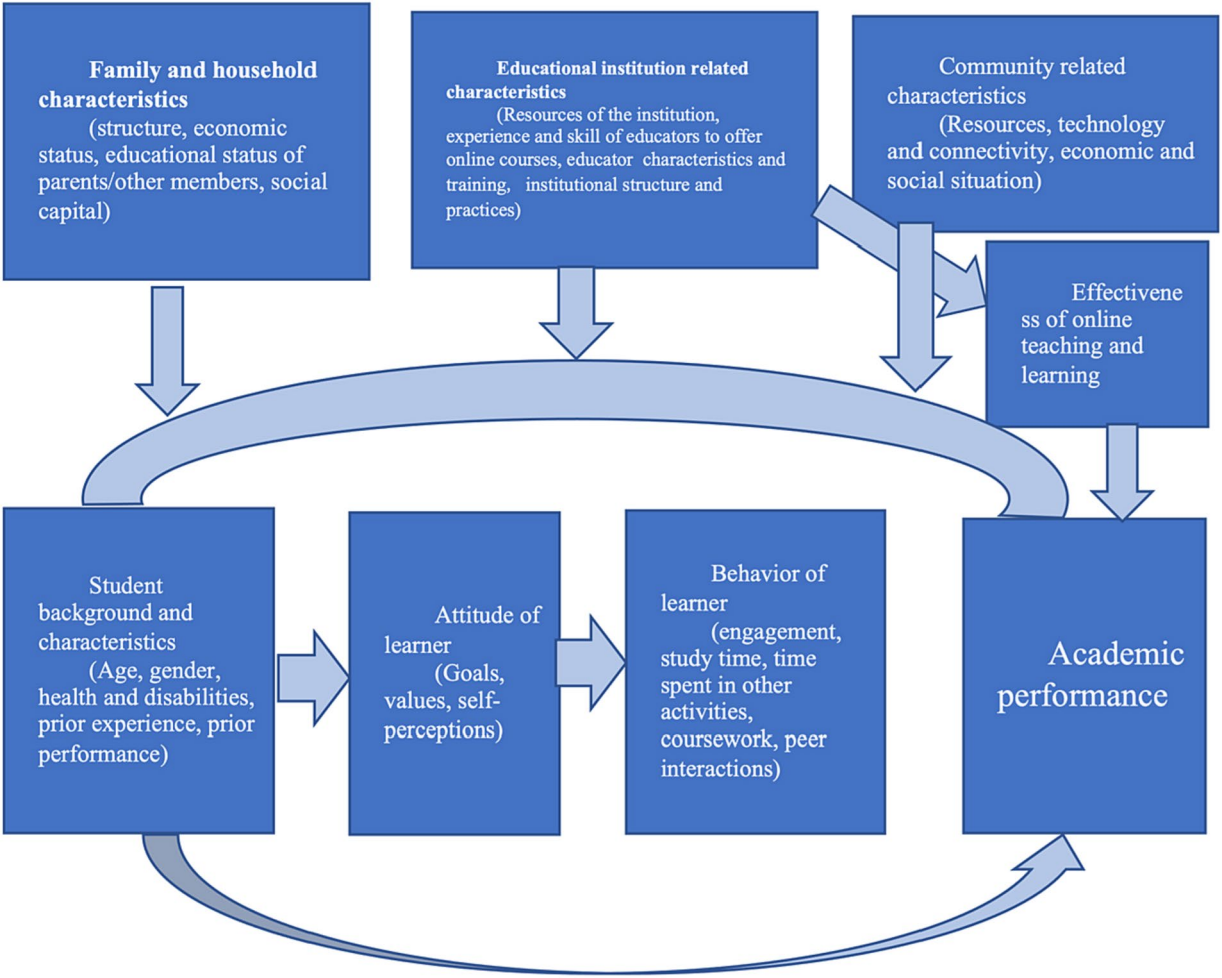
Conceptual framework to identify relevant factors affecting academic performance of college students.

This study was guided by an instructional framework for emergency remote teaching (Hodges et al., 2020; Rubtsova et al., 2023) as well as a generalized model of student learning and performance (Moore et al., 2014). The framework help identification of potential factors affecting student performance in general and in emergency remote teaching. The change in the mode of delivery of teaching material during COVID affected students’ learning (Cranfield et al., 2021). The academic success of students in online environment should depend on various individual, household, and community-level variables as well as student specific characteristics. The ‘major’ or discipline of the student may also be important as online teaching may not be equally effective for all subject areas. Absence of in-person interactions significantly alter the customary approach of learning and acquiring skills. Since the teaching materials must be delivered online, the experiences and knowledge of the educators about online teaching platforms become one of the most important factors affecting quality of online instruction and students’ learning. A conceptual framework has been presented in Figure 1. The purpose of the conceptual model is to help identify the potential factors affecting learning, not necessarily showing all possible interactions among the variables. The conceptual model has been modified to emphasize online teaching and learning and for students at the higher educational institutions (Fenollar et al., 2007; Alhadabi and Karpinski, 2020; Zach and Yazdi-Ugav, 2021; Al-Tameemi et al., 2023).

It should be noted that some of the variables in the conceptual model may not be available in the data sets publicly available, but this type of generalized framework will be useful to ensure that all relevant variables are considered in the empirical analysis. Our main objectives are to examine the effect of time spent on studying, time spent on gaming and other online activities, and health status of students on student performance.

### 2.2. Participants

The data used in this study were obtained from the 2020 China Family Panel Studies (CFPS), conducted by the Institute of Social Science Survey of Peking University. The CFPS is a general-purpose, nationally representative, longitudinal survey. The survey sample was drawn from twenty-five provinces and their administrative equivalents; thus, it represents 95% of the total population in the Chinese mainland. A multistage probability sampling proportional to size was used for the survey. More details about the CFPS are available in Xie and Hu (2014).

The CFPS primarily conducts face-to-face interviews. When the CFPS fails to complete face-to-face interviews, telephone interviews or web-based interviews are used as a substitute. The CFPS respondents are reinterviewed every two years, with the first wave in 2010 and five follow-ups happened during 2012, 2014, 2016, 2018, and 2020. Due to the COVID-19 pandemic, the CFPS primarily conducted telephone interviews, and about 89% of respondents were interviewed by telephone. 554 interviewers from all over the country worked for 176 days to improve the response rate of telephone-based surveys (Institute of Social Science Survey of Peking University, 2022). The 2020 CFPS had a total sample of 28,530 individuals. Only the individuals who were enrolled in a three-year of college or undergraduate college at the time of the interview were selected for this study. The final sample consisted of 728 individuals. Nationally, 41.8 million college students represented 2.97 percent of the Chinese population in 2020 (Ministry of Education of the People’s Republic of China, 2021). This study’s proportion of college students was 2.55%, which is closer to the Ministry of Education’s estimate. Even though the sample is a small proportion of total individuals surveyed, the absolute size of the sample is not very low and more than 700 can be considered sufficient for the further analyses.

### 2.3. Measures

#### 2.3.1. Academic performance

The principal objective of the study is to explain academic performance of students during the COVID period. For empirical analysis, a variable reflecting academic performance was used. Academic performance in the survey has been defined as an ordered categorical variable with values ranging from 1 to 5, 1 indicating academic performance at the top 10% level, 2 indicating the placement of students in between the top 11–25% of students, 3 when academic performance is in between the top 26–50%, 4 in between the top 51–75%, and 5 when performance falls within the bottom 24% of students. The CFPS used the following question to obtain the information: ‘What was your rank in your major in the academic calendar spring semester 2019–2020?’. A five-point Likert scale to measure of academic performance has been suggested by several previous studies (Chen et al., 2021; Yang et al., 2022).

#### 2.3.2. Health status

This study assessed health status of students using physical and mental health situations. Physical health was measured by the question, ‘During the past two weeks, have you felt any physical discomfort?’, and yes was coded as 1, and no was coded as 0. The 8-item Center for Epidemiologic Studies Depression Scale (CESD-8) was used to assess mental health. In the CFPS, each respondent was asked, ‘How often have you felt or behaved this way during the last week?’. The survey consists of 8 items (e.g., I felt depressed, my sleep was restless, and I was happy), which can be rated on a 4-point Likert scale from 0 (less than 1 day) to 3 (5–7 days). The range of the CESD-8 total score is 0 to 24, with high scores indicating higher level of depressive symptoms. The CESD-8 has shown reasonable validity and reliability as a measure of mental health among Chinese university students (Jiang et al., 2019).

#### 2.3.3. Internet use

Internet use included the use of the Internet for academic purposes (E-learning) and entertainment purposes (online gaming). In the CFPS, each respondent was asked, ‘Have you ever taken online courses, like Massive Open Online Courses (MOOCs), in the last week (except for regular college courses)?’ Respondents who reported ‘Yes’ were then asked. ‘Do you take online courses every day in the last week (except for regular college courses)?’. The outcome variable, E-learning, coded as 1 ‘never’, 2 ‘some days’, and 3 ‘every day’. In addition, online gaming was grouped into three levels: never, some days, and every day. The questions in the CFPS that collected information on online gaming were: ‘In the last week, did you play online games?’ and ‘Did you play online games every day in the last week?’. Internet use is the key independent variable. In this study, we considered both Internet use patterns (E-learning and online gaming) and Internet use status (use of Internet and intensity of Internet use). This definition is consistent with previous studies (Yang et al., 2023; Zheng et al., 2023).

#### 2.3.4. Study time

This study measured study time using total hours of study time in a week, which is a continuous variable. The CFPS questions related to this variable were “In general, how many hours did you spend on studying college work during weekdays?” and ‘In general, how many hours did you spend on studying college work on weekends?’

#### 2.3.5. Covariates

Consistent with the conceptual framework, the study included several individual, household, and area-related variables. The following covariates were selected to explain academic performance: male, age, rural residency, mothers’ educational attainment, fathers’ educational attainment, public college, and household education expenditure. The definitions of the variables are provided in Table 1.

**Table 1:** Definitions of the variables used in the empirical models.

Variable	Description	Source	Mean (Std)	%
Academic performance		CFPS adult questionnaire	2.35 (0.98)	
Level 1	Top 10%			22.44
Level 2	Between 11–25%			32.31
Level 3	Between 26–50%			34.26
Level 4	Between 51–75%			9.59
Level 5	Bottom 24%			1.40
Physical health	Physical discomfort in the prior 2 weeks	CFPS adult questionnaire		13.42
Mental health	Center for Epidemiologic Studies Depression Scale (CESD-8), individual sum score is 0 to 24.	CFPS adult questionnaire	12.29 (3.26)	
E-learning		CFPS adult questionnaire		
Never	The individual never participated in online courses in the last week			44.14
Some days	The individual participated in online courses some days in the last week			28.88
Every day	The individual participated in online courses every day in the last week			26.98
Online gaming		CFPS adult questionnaire		
Never	The individual never played online games in the last week			41.31
Some days	The individual played online games some days in the last week			35.84
Every day	The individual played online games every day in the last week			22.85
Study time	Total hours of study time in a week	CFPS adult questionnaire	9.99 (0.19)	
Public college	The school that the college students are currently attending is public school	CFPS adult questionnaire		72.13
Sociodemographic factors				
Male	The individual was male	CFPS adult questionnaire		47.02
Age	Actual age in years	CFPS adult questionnaire	21.49 (3.75)	
Rural residency	The individual was rural resident	CFPS adult questionnaire		41.52
Mothers’ educational attainment	The individual’s mother graduated from an above three-year college	CFPS adult questionnaire		7.67
Fathers’ educational attainment	The individual’s father graduated from an above three-year college	CFPS adult questionnaire		9.69
Household education expenditures	The education expense directly paid by the family during the past year in yuan.	CFPS family questionnaire	14,292.22 (10,777.98)	
Majors		CFPS adult questionnaire		
Medicine and health science	The individual’s major was medicine and health science			11.97
Energy resources, manufacturing, and civil engineering	The individual’s major was energy resources, manufacturing, or civil engineering hydraulics			29.82
Traffic and transport	The individual’s major was traffic and transport			36.52
Information technology, commerce, others	The individual’s major was information technology, commerce, or others			21.69

### 2.4. Multiple imputation of missing values

The median non-response rate for the 2020 CFPS was 7.22% (ranging from 6.18 to 33.79%) among the variables used in this study. For these missing values, we adopted the missing at random assumption (Little’s missing completely at random test and logit models were employed, data are not shown). Under the missing at random assumption mechanism, the probability of a missing value for an item may depend on observed data but not on unobserved data (Rubin, 1976). Multiple imputation allows researchers to increase the availability of data points, thus reducing biases when observations with missing data are deleted (Penn, 2007). Multiple imputation has three elemental phases: imputation, analysis, and pooling. The imputation phase was to create 50 copies of the dataset in this study, with the missing values replaced by imputed values using multiple imputation by chained equations (MICE). The MICE is a practical approach to impute missing data in multiple variables based on a set of univariate imputation models (White et al., 2011). The variables listed in Table 2 were used in the imputation models. We also included many auxiliary variables (smoking, drinking, physical inactivity, and intelligence quotient). Each of the 50 complete datasets was analyzed using a desired statistical method in the analysis phase. The results obtained from 50 completed datasets were combined into a single multiple-imputation result in the pooling phase. We used Rubin’s combination rules to obtain the estimates from multiple imputed data. The multiple imputation point estimate is the average of 50 regression coefficient estimates from the imputed datasets. Moreover, the multiple imputation estimate of the standard errors (SEs) are calculated based on within imputation variance and the between imputation variance.

**Table 2:** Variables used for the analysis of academic performance, 2020 CFPS.

Variable	Complete response, *N*	Complete %	Missing response, *N*	Missing %
Academic performance	482	66.21	246	33.79
Demographics and Social determinants				
Male	683	93.82	45	6.18
Age	728	100.00	0	0
Rural residency	600	82.42	128	17.58
Mothers’ educational attainment	594	81.59	134	18.41
Fathers’ educational attainment	588	80.77	140	19.23
Household education expenditures	682	93.68	46	6.32
Majors	673	92.45	55	7.55
Health status				
Physical health	678	93.13	50	6.87
Mental health	679	93.27	49	6.73
Internet use				
E-learning	674	92.58	54	7.42
Online gaming	674	92.58	54	7.42
Study time	680	93.41	48	6.59
Public college	677	92.99	51	7.01

### 2.5. Statistical analysis

A descriptive analysis of academic performance was performed by considering health status, Internet use, study time, and various individual characteristics. Statistical significance between groups was assessed through Pearson’s chi-square test for categorical variables and one-way ANOVA for continuous variables based on imputed data.

This study has used ordered probit regression models to analyze the effects of health, internet use, and study time on academic performance. We employed the tests (Wolfe-Gould, Brant, and Wald statistics) to evaluate the proportional odds assumption (Liu et al., 2023) implicit in the ordered probit regression. If all tests indicate that the proportional odds assumption is not valid, a generalized ordered probit model will be estimated as a robustness check, which allowed all coefficients to vary.

In the ordered probit regression models, the independent variables were health status (Model I), Internet use (Model II), and study time (Model III), respectively. The three models were adjusted for male, age, rural residency, mothers’ educational attainment, fathers’ educational attainment, public college, and household education expenditure. The final model included health status, Internet use, and study time simultaneously (Model IV). Preferred learning activities, and teaching presences (Lim and Richardson, 2022), the effects of health, internet use, and study time on academic performance may be varied across academic disciplines or majors. Stratification of students by area of specialization should be implemented to avoid potential bias created by differences in academic discipline or major. Therefore, the present study employed a sub-group analysis for medicine and health science, energy resources, manufacturing, and civil engineering, traffic and transport, and information technology, commerce, and others, respectively. Table 1 provides the list of the variables used in the analyses. The results are presented as coefficients (Coef.) along with their SEs and then the average marginal effects are calculated. All statistical analyses were conducted using Stata Version 17 (StataCorp, College Station, TX).

## 3. Results

The total sample size was 728 college students. A descriptive summary of selected variables for these respondents is shown in Table 1. The sample included 47.02% males and 41.52% living in rural areas. The average age of college students was 21.49 years. Academic performance was measured using a 5-point Likert scale; the average score was 2.35. The table also shows other health and related characteristics of the respondents. Physical discomfort was reported by 13.4% of respondents. Mental discomfort was also highly prevalent as shown by the sum score average for the CES-D 8 of 12.3.

**Figure 2:**
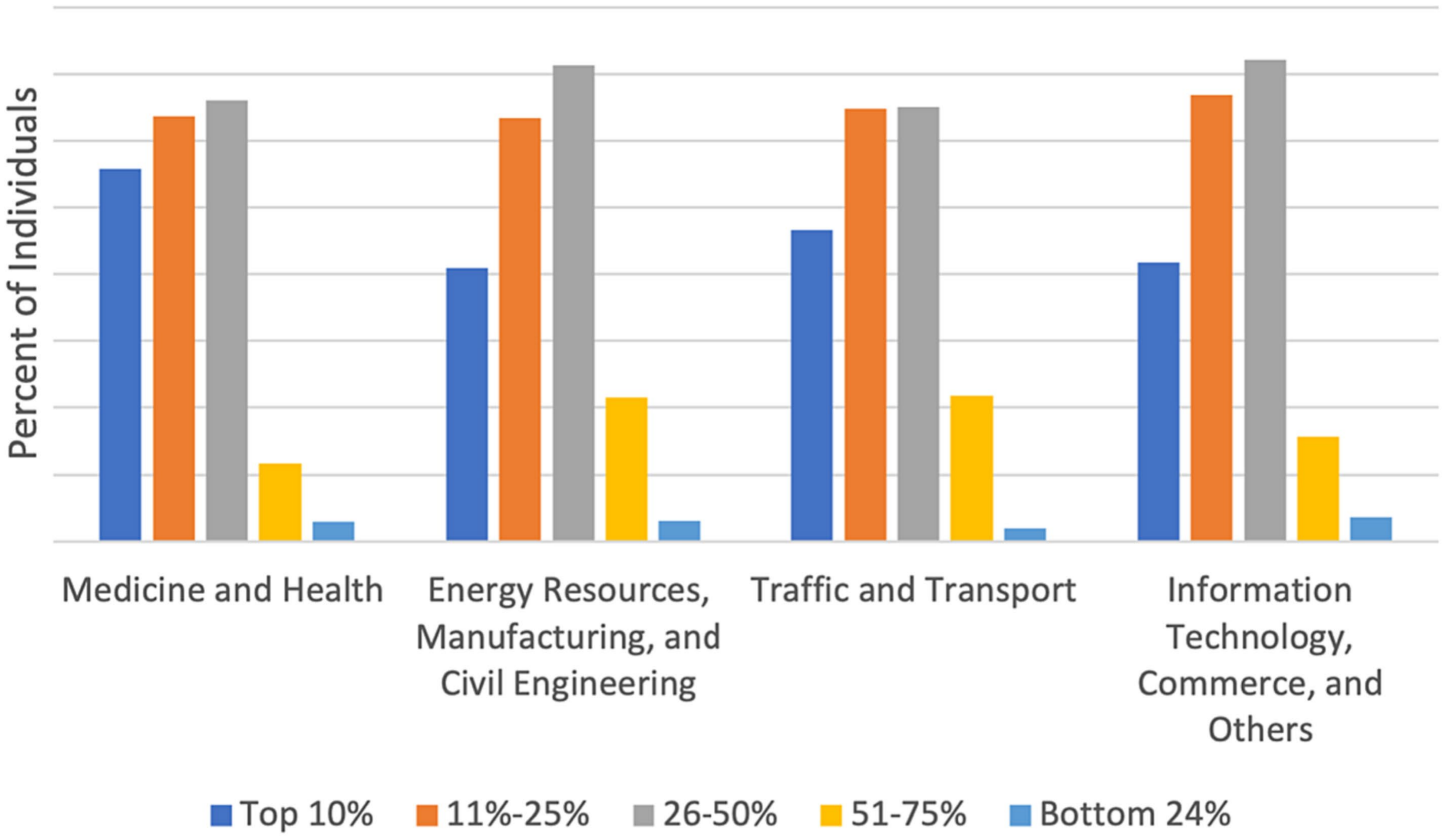
Percent distribution of students by academic performance categories (1–5 scale) for four broad academic disciplines or majors.

Figure 2 shows the distribution of academic performance on the 1–5 scale by four major categories (medicine and health science, energy resources, manufacturing, and civil engineering, traffic and transport, and information technology, commerce, and others). The distribution of academic performance is skewed to the right, with most college students reporting academic performance in the range of 1 to 3. Moreover, approximately 45% of college students reported low or medium level of academic performance (in the range of 3 to 5).

Table 3 shows the differences on the levels of academic performance according to health status, Internet use, study time, and various individual characteristics. The results of univariable analyses indicated that there were significant differences in college students’ academic achievement across Internet use and study time.

**Table 3:** Academic performance and related covariates, 2020 China Family Panel Studies (CFPS), *N* = 728.

	Performance categories				
	Level 1	Level 2	Level 3	Level 4	Level 5
Physical health (%)^#^	12.12	14.16	11.83	15.38	20.00
Mental health (mean)	12.02	12.29	12.35	12.41	13.86
Internet use (%)					
E-Learning (some days or every days)*	61.46	59.38	51.88	47.79	35.60
Online gaming (some days or every days)**	45.85	54.16	65.61	76.64	78.17
Study time, hours (mean)**	11.30	10.49	9.00	9.18	7.28
Public college (%)	75.87	72.93	69.96	71.70	52.71
Majors (%)					
Medicine and health science	14.85	11.79	11.54	7.32	12.90
Energy resources, manufacturing, civil engineering	27.17	29.22	31.01	33.42	32.08
Traffic and transport	37.84	36.59	34.63	41.48	25.75
Information Technology, Commerce, Other	20.14	22.41	22.83	17.78	29.27
Sociodemographic factors					
Male (%)	42.85	43.45	50.05	54.21	74.26
Age (mean)	21.43	21.32	21.48	21.93	23.56
Household education expenditures (mean)	14,297.45	14,844.86	14,262.73	12,619.20	13,896.16
Rural residence (%)	39.61	44.42	41.16	38.77	33.40
Mothers’ educational attainment^#^ (%)	10.00	5.69	7.84	6.17	18.18
Fathers’ educational attainment (%)	10.67	8.97	8.98	11.96	13.20

^#^No observations in some imputations. To identify offending imputations, we run the command on individual imputations. Pearson’s chi-square test for categorical variables and one-way ANOVA for continuous variables. **Significantly different (*p* < 0.01); *Significantly different (*p* < 0.05).

Table 4 presents results from four statistical models. Each model estimates the association between academic performance and Health (Model I), patterns of internet engagement (Model II), total study time (Model III), or all factors combined (Model IV). In Model I, the coefficients of health status were adjusted for covariates. College students who reported physical discomfort were more likely to have poor academic performance, but the coefficient was not statistically significant (Coef. = 0.084, *p* > 0.1). College students with a higher depression score had greater odds of having poor academic performance, although not statistically significant (Coef. = 0.012, *p* > 0.1). In Model II, the coefficients of Internet use were adjusted for covariates. College students who took online courses some days were less likely to have poor academic performance than those who had never taken online courses (Coef. = −0.269, *p* < 0.05). However, college students who played online games (some days or every day) were more likely to have poor academic performance than those who had never played online games (Coef. = 0.432, *p* < 0.01; Coef. = 0.609, *p* < 0.01). In Model III, the coefficients of study time were adjusted for covariates. College students who spent more time studying on college work had lower probability of having poor academic performance (Coef. = −0.038, *p* < 0.05). In Model IV, we included all the key explanatory variables, and the coefficients of poor health, Internet use, and study time were adjusted for covariates. The Model IV results were very similar to Models I-III.

**Table 4:** Health status, Internet use, and study time on academic performance: results of ordered probit regression models.

	Model I	Model II	Model III	Model IV
	Coef. (SEs)	Coef. (SEs)	Coef. (SEs)	Coef. (SEs)
Physical health	0.084 (0.152)			0.121 (0.154)
Mental health	0.012 (0.014)			0.018 (0.014)
E-learning				
Never		Ref.		Ref.
Some days		−0.269 (0.127)**		−0.257 (0.128)**
Every day		−0.133 (0.125)		−0.074 (0.128)
Online gaming				
Never		Ref.		Ref.
Some days		0.432 (0.122)***		0.410 (0.124)***
Every day		0.609 (0.138)***		0.553 (0.140)***
Study time			−0.038 (0.011)**	−0.031 (0.012)**
Male	0.193 (0.100)*	0.001 (0.111)	0.173 (0.101)*	0.012 (0.112)
Age	−0.002 (0.019)	0.022 (0.019)	−0.009 (0.019)	0.011 (0.020)
Rural residency	−0.014 (0.108)	−0.001 (0.110)	0.004 (0.108)	0.014 (0.109)
Mothers’ educational attainment	−0.251 (0.229)	−0.262 (0.233)	−0.273 (0.234)	−0.279 (0.235)
Fathers’ educational attainment	0.147 (0.202)	0.197 (0.203)	0.219 (0.209)	0.238 (0.207)
Public college	−0.118 (0.129)	−0.072 (0.133)	−0.076 (0.131)	−0.055 (0.135)
Household education expenditures (log)	−0.050 (0.027)*	−0.052 (0.028)*	−0.042 (0.027)	−0.041 (0.028)
Observations	728	728	728	728

***Indicates statistical significance at the 1% level, ** at the 5% level, and * at the 10% level.

The sign of coefficients shows whether the latent variable increases with the independent variable. To obtain predicted probabilities, this study employed marginal effects to measure the effect size for the ordered probit model. The results of the average marginal effects of health status, internet use, and study time on academic performance are presented in Appendix 1. College students who took online courses some days were 7.4% more likely to be at the top 10% of academic performance than those who had never taken online courses. Compared to those who had never played online games, college students who played online games some days (every day) were 12.2% (15.6%) less likely to be at the top 10% of academic performance. College students who spent more time studying on college work had a 0.9% increased likelihood of being at the top 10% of academic performance. The predicted probability for each of the values of the variable specified can similarly be explained.

The results of the ordered probit models, stratified by four major categories, are reported in Table 5. Medicine and health science students who played online games every day were more likely to have poor academic performance than those who had never played online games (Coef. = 1.277, *p* < 0.05). Similar results were obtained among college students in traffic and transport majors. College students in energy resources, manufacturing, and civil engineering majors who played online games (some days or every day) were more likely to have poor academic performance than those who had never played online games (Coef. = 0.779, *p* < 0.01; Coef. = −0.794, *p* < 0.01). Medicine and health science students who spent more time studying on college work had lower probability of having poor academic performance (Coef. = −0.069, *p* < 0.1). College students in traffic and transport majors who took online courses some days were less likely to have poor academic performance than those who had never taken online courses (Coef. = −0.446, *p* < 0.05).

**Table 5:** Effects of health status, Internet use, and study time on academic performance by discipline or major: results of ordered probit regression models.

	Medicine and health science	Energy resources, manufacturing, and engineering	Traffic and transport	Information technology, commerce, and others
	Adjusted Coef. (SEs)	Adjusted Coef. (SEs)	Adjusted Coef. (SEs)	Adjusted Coef. (SEs)
Physical health	0.179 (0.435)	0.160 (0.285)	−0.003 (0.223)	0.176 (0.393)
Mental health	0.024 (0.064)	0.030 (0.024)	0.025 (0.024)	−0.022 (0.035)
Online courses				
Never	Ref.	Ref.	Ref.	Ref.
Some days	−0.223 (0.337)	−0.131 (0.227)	−0.446 (0.215)**	−0.276 (0.265)
Every day	0.026 (0.438)	0.038 (0.237)	−0.327 (0.208)	0.075 (0.281)
Online games				
Never	Ref.	Ref.	Ref.	Ref.
Some days	0.149 (0.365)	0.776 (0.224)***	0.300 (0.219)	0.253 (0.282)
Every day	1.267 (0.609)**	0.790 (0.243)***	0.496 (0.257)*	0.237 (0.233)
Study time	−0.069 (0.036)*	−0.031 (0.020)	−0.030 (0.028)	−0.032 (0.029)
Observations	87	217	266	158

Adjusted for male, age, rural residency, mothers’ educational attainment, fathers’ educational attainment, public college, and household education expenditure. ***Indicates statistical significance at the 1% level, ** at the 5% level, and * at the 10% level.

This study employed the Wolfe-Gould, Brant, and Wald statistics to evaluate the proportional odds assumption. All the tests suggest that the proportional odds assumption is unlikely to hold for the data (data are not shown). The results of the generalized ordered probit model are reported in Table 6. The generalized ordered probit model consists of four underlying binary dependent variable equations. The first model estimates the relative effects of independent variables on academic performance category 1 (top 10%) vs. 2 (between 11–25%) to 5 (bottom 24%), and the second model estimates the coefficients for academic performance categories 1 (top 10%) to 2 (between 11–25%) vs. 3 (between 26–50%) to 5 (bottom 24%), and so on, so forth. The results of the generalized ordered probit model are similar to those of the ordered probit models and can be interpreted the same way.

**Table 6:** Health status, Internet use, and study time on academic performance: results of generalized ordered probit regression models.

	Model I	Model II	Model III	Model IV
	1 vs. 2–5	1–2 vs. 3–5	1–3 vs. 4–5	1–4 vs. 5
	Coef. (SEs)	Coef. (SEs)	Coef. (SEs)	Coef. (SEs)
Physical health	0.061 (0.198)	0.191 (0.180)	0.127 (0.232)	−2.492 (2.700)
Mental health	0.022 (0.019)	0.014 (0.017)	0.014 (0.024)	0.119 (0.214)
E-learning				
Never	Ref.	Ref.	Ref.	Ref.
Some days	−0.185 (0.155)	−0.234 (0.149)	−0.402 (0.225)*	−0.107 (1.771)
Every day	−0.031 (0.161)	−0.082 (0.156)	−0.066 (0.199)	−0.680 (1.068)
Online gaming				
Never	Ref.	Ref.	Ref.	Ref.
Some days	0.347 (0.160)**	0.433 (0.147)***	0.581 (0.219)***	0.694 (1.992)
Every day	0.562 (0.184)***	0.527 (0.167)***	0.635 (0.238)***	1.651 (2.570)
Study time	−0.032 (0.014)**	−0.044 (0.014)***	−0.009 (0.021)	−0.014 (0.092)
Male	−0.040 (0.146)	0.034 (0.131)	0.038 (0.175)	0.207 (0.816)
Age	−0.006 (0.024)	0.006 (0.022)	0.039 (0.030)	0.088 (0.146)
Rural residency	0.109 (0.142)	−0.014 (0.137)	−0.096 (0.179)	0.361 (0.841)
Mothers’ educational attainment	−0.338 (0.269)	−0.082 (0.263)	−0.419 (0.400)	−2.144 (3.223)
Fathers’ educational attainment	0.207 (0.252)	0.128 (0.245)	0.336 (0.302)	0.562 (1.079)
Public college	−0.122 (0.171)	−0.026 (0.722)	0.086 (0.231)	−0.601 (0.917)
Household education expenditures (log)	−0.055 (0.042)	−0.032 (0.033)	−0.021 (0.045)	−0.069 (0.192)
Observations	728			

***Indicates statistical significance at the 1% level, ** at the 5% level, and * at the 10% level.

We restricted the sample to those who were enrolled in a three-year of college or undergraduate college at the time of the interview. Therefore, the sample size is relatively small, especially for the sub-group analysis. While the selected sample cannot be relied on for a larger population of college students in China, this unrepresentativeness does not necessarily affect the generalizability of findings about relationships between variables (Manion, 1994).

## 4. Discussion

The main objectives of the study are to examine effects of health status, Internet use, and study time on academic performance of Chinese college students during the COVID-19 pandemic. Since the academic performance is defined in a relative way, as expected, about 45% of college students reported low or medium level of academic performance in China during the COVID-19 pandemic. Low level of academic performance needs to be approached more closely by educators. We need to identify these high-risk students early in their journey to prevent this low performance. More importantly, good academic performance can produce high-quality graduates who will become good leaders and enhance quality human resources for the country (Ali et al., 2009). Therefore, it is important for policymakers to design effective interventions for improving students’ academic performance.

Our results indicate that Internet use was significantly correlated with academic performance. Internet use for academic purposes showed decreased odds of having poor academic performance during the COVID-19 pandemic. Since the beginning of the COVID-19 in China, 1,454 colleges and universities across the country have transitioned to online teaching, and more than 950,000 teachers offered 942,000 online courses (Ministry of Education of the People’s Republic of China, 2020). An accumulative number of 1.18 billion person-times that Chinese college students have participated in online courses. At the same time, the number of online MOOCs increased by 5,000, and the total number reached 23,000 by 3 April 2020 (Ministry of Education of the People’s Republic of China, 2020). College students can attend live-streamed classes for regular college courses, while they can also use the Internet for academic purposes, like MOOCs, to enhance learning (Abhishek et al., 2023).

This study found that Internet use for entertainment increased the probability of having poor academic performance during the COVID-19 pandemic. With the lockdown and social distancing during the outbreak of COVID-19, internet use and gaming of adolescents increased, which may have affected online learning of academic subject areas. Although Internet use and gaming are alternative leisure activities during lockdowns, they may also increase the risk of internet addiction and participation in problematic gaming (Wu et al., 2022a,b). Previous studies have shown that internet addiction and being participants in problematic games adversely affect academic performance (Akhter, 2013; Islam et al., 2020; Elbilgahy et al., 2021). This study is valuable because it makes the current workforce of teachers aware of the threats and opportunities of online gaming. This recreational activity competes with study time. However, features of online gaming make it appealing to a subset of learners. Closer study of online gaming and features that make it attractive can improve teaching across several subject areas.

We found that college students who spent more time studying on college work were less likely to have poor academic performance. The unfolding of the COVID-19 pandemic has changed students’ learning habits and routines and allowed for greater student autonomy (Anthonysamy and Singh, 2023). Students have more time to study and review study material in online learning environment (Amir et al., 2020). Previous studies have shown that study time is positively associated with good academic performance (Ralph and Stinebrickner, 2008; Liu, 2022). Educators need to develop strategies for teaching students about the need to avoid online gaming while doing their E-learning courses. Teaching time management is an unfamiliar skill to teachers. In the digital era, it is as vital as learning to read.

Poor health was not found significant in explaining academic performance probably because of difficulty of measuring health status of individuals, especially for younger age group college students. As the descriptive analysis indicates, percent of students reporting physical and mental health concerns were relatively low, which may have affected statistical significance of the parameters for relatively small sample size of college students.

The sub-group analysis for four major categories found that associations between internet use for entertainment purposes and academic performance were significant for medicine and health science, traffic and transport, and energy resources, manufacturing, and civil engineering students but not for other majors (information technology, commerce, and others). Therefore, the association between internet use for entertainment purposes and academic performance is not straightforward and vary according to academic discipline of the student. Learning goals and approaches for competency development are different for different academic disciplines or majors. For example, disciplines such as medicine and health science emphasize knowledge application and experience in practice settings. Discipline-specific learning goals, competencies to be achieved also affect student motivation, values and self-perception and combining all disciplines together dilutes the effects of study time, internet use and health status on academic performance. Therefore, students’ academic performance can be better explained by discipline-specific analysis (Breen and Lindsay, 2002). However, more rigorous discipline-specific analysis could not be carried out due to relatively small sample size of college students in the data set.

### 4.1. Implications

Policymakers can help improve educational outcomes and address educational inequalities among college students by considering a series of reforms. The present study has identified several factors affecting academic performance, and some of the factors are amenable to policy changes. First, to tackle future pandemics, the Chinese government should allocate more resources to universities to improve preparedness for offering effective online learning platforms. Although, we do not have direct evidence on this, relatively poor performance of students indicates lack of availability of quality online courses. Second, internet addiction and playing problematic games affect academic performance significantly in some specific academic disciplines. Educational attainment of students can be improved through treatment and counseling of internet misuse. Students can be advised on how to self-identify internet addiction and strategies to avoid problematic use of internet or games. Third, college students should receive trainings on time management skills that will enable them to use available time in an efficient manner and improve effective learning in an online learning environment. However, the major barriers to policy implementation are lack of funding and resources, lack of school psychologists, and lack of awareness of time management.

### 4.2. Limitations

Although the current study used a national survey to analyze factors affecting college students’ academic performance in China during the COVID-19 pandemic, several limitations of the study should be noted. First, this cross-sectional study is based on 2020 CFPS during the COVID-19 pandemic and the pandemic itself may have affected the quality of data collected. Since the study is based on data collected from a cross-section of college students, no causal inferences can be derived. A useful direction for future research would be to use longitudinal survey information on changes in academic performance due to a switch from one mode of delivering course materials to another (such as, in-person to online). Second, The CFPS survey does not collect information on study motivation, study strategy, and instructors’ characteristics, so this study could not include these factors as possible covariates. Previous studies have shown a significant positive relationship between motivation and academic performance. Study strategy is a predictor of students’ academic performance. Instructors’ characteristics strongly determine students’ academic performance (Al-Tameemi et al., 2023) but none of these variables are available in our data set. Another limitation is the sample size of college students in the CFPS data set. Although the college student proportion in total sample is similar to proportion of population attending college, the sample size was less than eight hundred and the estimators may not be particularly precise. Last, the data were obtained via survey, and thus the limitations of all self-reported data exist, such as recall bias and the unreliability of responses when respondents are under pressure.

## 5. Conclusion

Greater understanding of factors affecting academic performance during the COVID-19 pandemic will help design effective interventions for improving academic achievements. This study examined the factors (health status, Internet use, and study time) associated with academic performance among Chinese college students during the COVID-19 pandemic. The empirical findings indicate that Internet use for academic purposes improve student academic performance while spending too much time on the internet and playing online games lowered performance. Physical and mental health status show positive effects on academic outcomes although the coefficients are not statistically significant. This may be due to relatively small sample size. In future research, combining multiple years of data may help overcome this problem.

In any case, the current study identified several relevant variables that have affected student’s academic performance during the pandemic. Therefore, the study adds to the literature on the association between internet use/study time and academic performance. The cross-sectional study, however, does not establish causality between the identified factors and academic performance. Given the availability of information in the survey, the study has adjusted for a wide variety of individual, household, and area-related variables but, it is still possible that unmeasured confounders may explain the current findings. It is interesting to note that educational attainment of parents or educational expenditure of households had no statistically significant effect on academic performance implying that student-specific factors are more important.

These results can be used to design policies to improve educational outcomes and to address educational inequalities. The colleges should improve preparedness for offering effective online learning platforms. College students should also receive help or training on effective time management, educational strategies without direct and continuous supervision and use and misuse of Internet resources.

## Data availability statement

The original contributions presented in the study are included in the article/supplementary material, further inquiries can be directed to the corresponding author.

## Ethics statement

Ethical review and approval was not required for the study on human participants in accordance with the local legislation and institutional requirements. Written informed consent from the patients/participants OR patients/participants legal guardian/next of kin was not required to participate in this study in accordance with the national legislation and the institutional requirements.

## Author contributions

CL: Conceptualization, Formal analysis, Methodology, Writing – original draft. LK: Conceptualization, Funding acquisition, Methodology, Writing – review & editing. TM: Supervision, Writing – review & editing. MK: Supervision, Writing – review & editing.

## Appendix 1

Marginal effects of the ordered probit model.

**Table:**

	Academic performance				
	Level 1	Level 2	Level 3	Level 4	Level 5
	dy/dx (SEs)	dy/dx (SEs)	dy/dx (SEs)	dy/dx (SEs)	dy/dx (SEs)
Physical health	−0.034 (0.043)	−0.011 (0.014)	0.023 (0.030)	0.017 (0.023)	0.004 (0.005)
Mental health	−0.005 (0.004)	−0.002 (0.001)	0.003 (0.003)	0.003 (0.002)	0.001 (0.001)
Online courses					
Never	Ref.	Ref.	Ref.	Ref.	Ref.
Some days	0.074 (0.037)**	0.021 (0.011)*	−0.052 (0.027)*	−0.035 (0.002)**	−0.008 (0.005)*
Every day	0.020 (0.035)	0.008 (0.014)	−0.014 (0.024)	−0.011 (0.019)	−0.003 (0.005)
Online games					
Never	Ref.	Ref.	Ref.	Ref.	Ref.
Some days	−0.122 (0.037)***	−0.032 (0.012)***	0.088 (0.028)***	0.054 (0.018)***	0.011 (0.005)**
Every day	−0.156 (0.038)***	−0.052 (0.018)***	0.111 (0.028)***	0.079 (0.023)***	0.017 (0.008)**
Study time	0.009 (0.003)**	0.003 (0.002)**	−0.006 (0.002)**	−0.004 (0.002)**	−0.001 (0.001)*

Adjusted for male, age, rural residency, mothers’ educational attainment, fathers’ educational attainment, public college, and household education expenditure. ***Indicates statistical significance at the 1% level, ** at the 5% level, and * at the 10% level.
